# Estimated Glomerular Filtration Rate and Proteinuria Are Separately and Independently Associated with the Prevalence of Atrial Fibrillation in General Population

**DOI:** 10.1371/journal.pone.0079717

**Published:** 2013-11-06

**Authors:** Yoshiaki Ohyama, Masahiko Imai, Masahiko Kurabayashi

**Affiliations:** 1 Department of Medicine and Biological Science, Gunma University Graduate School of Medicine, Maebashi, Gunma, Japan; 2 Clinical Investigation and Research Unit, Gunma University Hospital, Maebashi, Gunma, Japan; 3 Department of Cardiology, Gunma Chuo General Hospital, Maebashi, Gunma, Japan; Innsbruck Medical University, Austria

## Abstract

**Background:**

Both, proteinuria and a decline in glomerular filtration rate (GFR) are associated with greater cardiovascular mortality. However, few studies have explored that proteinuria and lower GFR are related with prevalent atrial fibrillation (AF).

**Methods:**

This cross-sectional study was based on annual health check-up program of community-based population in Gunma, Japan from April 2011 to March 2012. A total of 20,019 adult participants were included. AF was ascertained by a standard 12-lead electrocardiogram. Cross-sectional association and correlates with prevalent AF were examined using multivariable logistic regression analysis.

**Results:**

The overall prevalence of AF was 0.6% (2.2 % in participants with eGFR < 60 mL▪min^-1^･1.73m^-2^, 0.4% and 0.2% in those with eGFR 60 to 89 and ≧90 mL▪min^-1^･1.73m^-2^, p for trend <0.001). The multivariable odds ratio (OR) for AF was 2.86 (95 % CI 1.16 - 7.08, p<0.001) for eGFR< 60 mL▪min^-1^▪1.73m^-2^ versus eGFR≧ 90 mL▪min^-1^▪1.73m^-2^. This association remained significant with further adjustment for proteinuria. In addition, proteinuria was also strongly associated with increased prevalence of AF (OR 2.96, 95 % CI 1.55-5.68, p<0.001), an association that remained significant after adjustment for eGFR.

**Conclusions:**

Proteinuria and lower eGFR are separately and significantly associated with prevalence of AF independent of well-established risk factors for AF in general population.

## Introduction

Atrial fibrillation (AF) is one of the most common arrhythmias that clinicians encountered in general practice. Because AF predisposes to ischemic stroke and cardiac death, an implementation of the primary preventive measures to the high-risk population for AF will be of paramount importance. Prior epidemiological studies established old age, hypertension, diabetes, male gender, and prevalent cardiovascular disease as some of the most important risk factors for non-valvular AF [1,2]. However, because those established risk factors do not fully explain the development of AF, further understanding of the prediction and pathophysiology of AF are required to help reduce the burden of AF in general population. . 

Chronic kidney disease (CKD), defined based on the combination of kidney damage (most often quantified using albuminuria) and reduced kidney function (quantified as glomerular filtration rate (GFR) estimated from serum creatinine concentration) [3], is highly prevalent and markedly increases the risk for cardiovascular events in the general population [4,5]. AF and CKD share many risk factors such as hypertension, diabetes, and preexisting cardiovascular disease [6,7]. While a high prevalence of AF has been demonstrated in end stage renal disease (ESRD), there are limited data on the prevalence and clinical correlates of AF among adults with less severe CKD, which is substantially more common than ESRD patients. Therefore, we examined the effect of 2 measures of kidney disease, reduced GFR and proteinuria, on the prevalence of AF in a large community-based population in Gunma, Japan.

## Methods

### Ethics

All subjects gave written informed consent, and all data from subjects were registered into the database of Gunma University. The protocol of the present investigation was approved by the ethic committee of Gunma University and was in accordance with the Declaration of Helsinki.

### Study subjects

Data from periodic health examinations of community residents and employees of companies and governments performed from April 2011 to March 2012 in Gunma Prefecture, Japan were collected. After obtaining approval of the study design from the ethics committee of the participating institute, the health examination data were collected from institute.

### Ascertainment of AF

AF was diagnosed electrocardiographically by a trained doctor in a participating center. Conventional diagnostic criteria of AF i.e., a grossly irregular ventricular rhythm of supraventricular origin, no visible P wave and irregular fluctuation of the baseline, were employed. In the present study, subjects were defined as having AF when their electrocardiogram showed AF at the time of health examination. Therefore, those with a history of paroxysmal AF but not having an AF episode at the time of the health examination were not counted as having AF in the following analysis.

### Measurement of Kidney Function

Kidney function was assessed 2 ways: level of estimated glomerular filtration rate (eGFR) based on creatinine and presence of proteinuria. eGFR (mL▪min^-1^▪1.73m^-2^) was calculated MDRD study equation modified for Japanese population: 194 × Serum creatinine^-1^.^094^ × Age^-0.287^ × 0.739 (if female). We categorized level of eGFR as follows: ≧90, 60 to 89, and <60 mL▪min^-1^▪1.73m^-2^. 

Proteinuria was defined as a urine dipstick protein result of ≧1+ in the absence of potential urinary tract infection (i.e., concomitant positive urine nitrite or esterase) found in laboratory databases. We excluded patients with prior kidney transplant. 

### Other clinical variables

 All subjects answered questions about medical history (hypertension, diabetes mellitus, dyslipidemia, cardiac disease, and liver disease) and smoking status and underwent physical examinations, including blood pressure, electrocardiography, and blood testing (serum creatinine, fasting glucose, HDL cholesterol, LDL cholesterol, triglyceride, and hemoglobin A1c). Body mass index was calculated as weight in kilograms divided by height in meters squared. Arterial blood pressure was carefully measured in the arm with the patient in a sitting position after resting for a few minutes. Cardiovascular risk factors were identified as (1) hypertension; use of antihypertensive agents, systolic blood pressure≧140 mmHg, or diastolic blood pressure ≧90 mmHg on admission; (2) diabetes mellitus; use of oral hypoglycemic agent or insulin, fasting blood glucose≧126mg/dl, random blood glucose ≧ 200 mg/dl, or glycosylated hemoglobin > 6.5%; (3) dyslipidemia; use of anti-hyperlipidemic agents or serum LDL-cholesterol ≧140 mg/dL and/or HDL-cholesterol < 40 mg/dL and/or triglyceride ≧ 150 mg/dL ; (4) smoking; any lifetime experience of cigarette use. Past and present illnesses were based on medical interviews of subjects by physicians. Cardiac disease included coronary heart disease and heart failure. 

### Statistical analysis

All analyses were carried out with SPSS. Frequency distributions of all variables were first inspected to identify anomalies and outliers possibly caused measurement artifacts. Continuous data were described by their mean and standard division (SD), and categorical data, as proportions (percentage). Differences in baseline characteristics between groups were determined by Student t test or analysis of variance for continuous variables (ANOVA) and by χ^2^ test for categorical variables. Multivariable logistic regression analysis was used to investigate the association between eGFR or proteinuria and prevalence of AF. All associations were first analyzed with adjustments for age and gender. A subsequent model included additional adjustment of other potential confounders, including hypertension, diabetes mellitus, smoking, and cardiac disease. We also examined for a possible interaction between eGFR level and proteinuria on the prevalence of AF in a separate multivariable model. A 2-tailed p<0.05 was considered significant. 

## Results

### Patient Characteristics

Total 20,019 subjects (mean age 53.2 years) were enrolled in this cross-sectional study. Table 1 shows selected characteristics of subjects by categories of eGFR and proteinuria. The mean eGFR among participants was 77.1 mL▪min^-1^･1.73m^-2^. In this study, older individuals were more likely to have lower eGFR and proteinuria. Hypertension, diabetes, dyslipidemia and a history of cardiovascular disease were more frequent among subjects with lower eGFR. Hypertension, diabetes, dyslipidemia and smoker were more frequent among subjects with proteinuria than those without proteinuria.

**Table 1:** Baseline Characteristics by Estimated Glomerular Filtration Rate and Proteinuria.

	eGFR, ml▪min^-1^▪1.73m^-2^					Presence of proteinuria		
	Total (n=20091)	≧90	60-89	<60	p Value	no proteinuria (n=19486)	proteiuria	p Value
		(n=3148)	(n=15212)	(n=1731)			(n=605)	
Men	12429 (62%)	1769 (56.2%)	9470 (76.2%)	1190 (68.7%)	<0.001	11964 (61.4%)	465 (76.9%)	<0.001
Age(y)	53.2 (9.2)	50.0 (8.3)	53.1 (8.9)	60.3 (9.9)	<0.001	53.2 (9.2)	55.8 (10.3)	<0.001
Hypertension	6122 (30.8%)	854 (27.1%)	4427 (29.1%)	841 (48.6%)	<0.001	5753 (29.5%)	369 (61.0%)	<0.001
Diabetes mellitus	1607 (8.0%)	347 (11.0%)	1056 (6.9%)	189 (10.9%)	<0.001	1398 (7.2%)	194 (32.1%)	<0.001
Dyslipidemia	5842 (29.5%)	770 (24.5%)	4382 (28.8%)	690 (39.9%)	<0.001	5549 (28.5%)	293 (48.4%)	<0.001
Smoker	5327 (26.5%)	1085 (34.5%)	3946 (25.9%)	296 (17.1%)	<0.001	5118 (26.3%)	209 (34.5%)	<0.001
Cardiac disease	638 (3.2%)	64 (2.0%)	448 (2.9%)	126 (7.3%)	<0.001	596 (3.1%)	42 (7.2%)	<0.001
Height(cm)	164.4 (8.7)	163.4 (8.9)	164.6 (8.6)	164.4 (8.6)	<0.001	164.3 (8.7)	165.8 (7.9)	<0.001
Weight (kg)	63.1 (12.2)	61.3 (12.5)	63.2 (12.1)	65.1 (12.0)	<0.001	62.9 (12.1)	69.2 (7.9)	<0.001
BMI	23.2 (3.4)	22.8 (3.6)	23.2 (3.3)	23.9 (3.2)	<0.001	23.2 (3.4)	25.1 (4.3)	<0.001
SBP	125.7 (16.2)	125.2 (16.1)	125.4 (14.1)	128.7 (16.4)	<0.001	125.4 (16.0)	134.2 (19.2)	<0.001
DBP	78.7 (10.8)	78.8 (10.8)	78.7 (10.8)	80.0 (10.9)	<0.001	78.6 (10.8)	82.5 (12.5)	<0.001
Atrial fibrillation	112 (0.56%)	6 (0.2%)	68 (0.4%)	38 (2.2%)	<0.001	97 (0.5%)	15 (2.5%)	0.003
Laboratory daga								
HDL	65 (17)	67.6 (18.9)	66.1 (17.7)	62.0 (17.2)	<0.001	66.1 (17.9)	60.4 (17.9)	<0.001
LDL	128 (32)	122.6 (33.3)	128.9 (31.9)	130.8 (31.9)	<0.001	128.0 (32.1)	130.7 (34.4)	<0.001
TG	118 (32)	113.5 (109.4)	117.4 (90.0)	129.1 (86.6)	<0.001	99.2 (20.5)	121.3 (44.7)	<0.001
Cre	0.77 (0.26)	0.60 (0.09)	0.77 (0.13)	1.10 (0.70)	<0.001	0.77 (0.21)	1.01 (0.89)	<0.001
eGFR	77.1 (14.2)	99.4 (11.8)	75.1 (7.7)	53.3 (7.6)	<0.001	77.3 (13.9)	68.9 (20.5)	<0.001
Glucose	99 (22)	103.6 (32.2)	98.8 (19.1)	102.0 (21.2)	<0.001	99.2 (20.5)	121.3 (44.7)	<0.001
HbA1c	5.3 (0.7)	5.4 (1.1)	5.3 (0.6)	5.4 (0.7)	<0.001	5.3 (0.7)	6.0 (1.4)	<0.001

Values expressed as number (percent) or mean (SD)

### Prevalence of AF

Among 20,019 subjects, 112 subjects (0.6 %) had prevalent AF. When stratified by kidney function, the prevalence was 0.2% among adults with eGFR≧90 mL▪min^-1^▪1.73m^-2^, 0.4 % among those with eGFR 60 to 89 mL▪min^-1^▪1.73m^-2^, and 2.2 % among those with eGFR <60 mL▪min^-1^▪1.73m^-2^ (Table 1). When stratified by proteinuria, the prevalence was 0.5 % among adults without proteinuria, and 2.5 % among those with proteinuria (Table 1).

Compared with subjects with eGFR≧90 mL▪min^-1^▪1.73m^-2^, the age- and gender-adjusted odds ratio (OR) for prevalent AF were 3.74 (95%CI, 1.54 to 9.10) for eGFR <60 mL▪min^-1^▪1.73m^-2^ (p<0.01, Table 2). The multivariable-adjusted OR for prevalent AF was slightly attenuated but remained statistically significant after adjustment for age, gender, hypertension, diabetes mellitus, smoking and cardiac disease. When this model was further adjusted for proteinuria, the association of eGFR with AF was essentially unaltered. 

**Table 2:** Odds Ratio (95%CI) for AF According to eGFR levels.

	eGFR, ml▪min^-1^▪1.73m^-2^		
	≧90	60-89 (n=15212)	<60 (n=1731)
	(n=3148)		
Cases of AF, n	6	68	38
Age-, gender-adjusted OR	1(ref)	1.62 (0.70-3.76)	3.74 (1.54-9.10)
(95% CI)			
Multivariable-adjusted* OR (95%CI)	1(ref)	1.53 (0.65-3.58)	2.86 (1.16-7.08)
Multivariable-adjusted* + proteinurea OR (95%CI)	1(ref)	1.55 (0.66-3.65)	2.66 (1.07-6.60)

* Adjusted for age, gender, hypertension, diabetes mellitus, smoking, and cardiac diasease

In addition, we evaluated the AF risk relationship with eGFR categorized by CKD-EPI (Chronic Kidney Disease Epidemiology Collaboration) equation modified for Japanese [8] instead of MDRD Study equation. Mean eGFR computed by the CKD-EPI equation was higher than by The MDRD Study equation, (81.8 vs 77.1 mL/min/1.73m2, respectively), and the prevalence of the impaired eGFR (<60 mL▪min^-1^▪1.73m^-2^) was lower in the CKD-EPI equation compared with that assessed by The MDRD Study equation (3.10% vs 8.72%, respectively). With respect to the relationships between eGFR and prevalent AF, similar finding were noted in both equations; the impaired eGFR (<60 mL▪min^-1^▪1.73m^-2^) subjects were associated with an increase in prevalent AF independent of age, gender, hypertension, diabetes mellitus, smoking, and cardiac disease. However, although a trend toward increased AF risk was noted in the impaired eGFR subjects, such an association did not remain significant after adjusting for proteinuria in addition to those variables (Table S1).

Subjects with proteinuria had approximately 3-fold increased risk of prevalent AF in the age-, gender-adjusted and multivariable-adjusted model. Further adjustment of eGFR had little effect on this association (Table 3). This association didn’t change after adjusted eGFR by CKD-EPI equation instead of that by MDRD study equation (Table S2). 

**Table 3:** Odds Ratio (95%CI) for AF According to Presence of Proteinuria.

	no proteinurea	proteinurea
	(n=19486)	(n=605)
Cases of AF, n	97	15
Age-, gender-adjusted OR	1(ref)	3.08 (1.75-5.43)
(95% CI)		
Multivariable-adjusted OR (95%CI)*	1(ref)	2.96 (1.55-5.68)
Multivariable-adjusted* + eGFR OR (95%CI)	1(ref)	2.67 (1.34-5.15)

* Adjusted for age, gender, hypertension, diabetes mellitus, smoking, and cardiac diasease.

**Figure 1:**
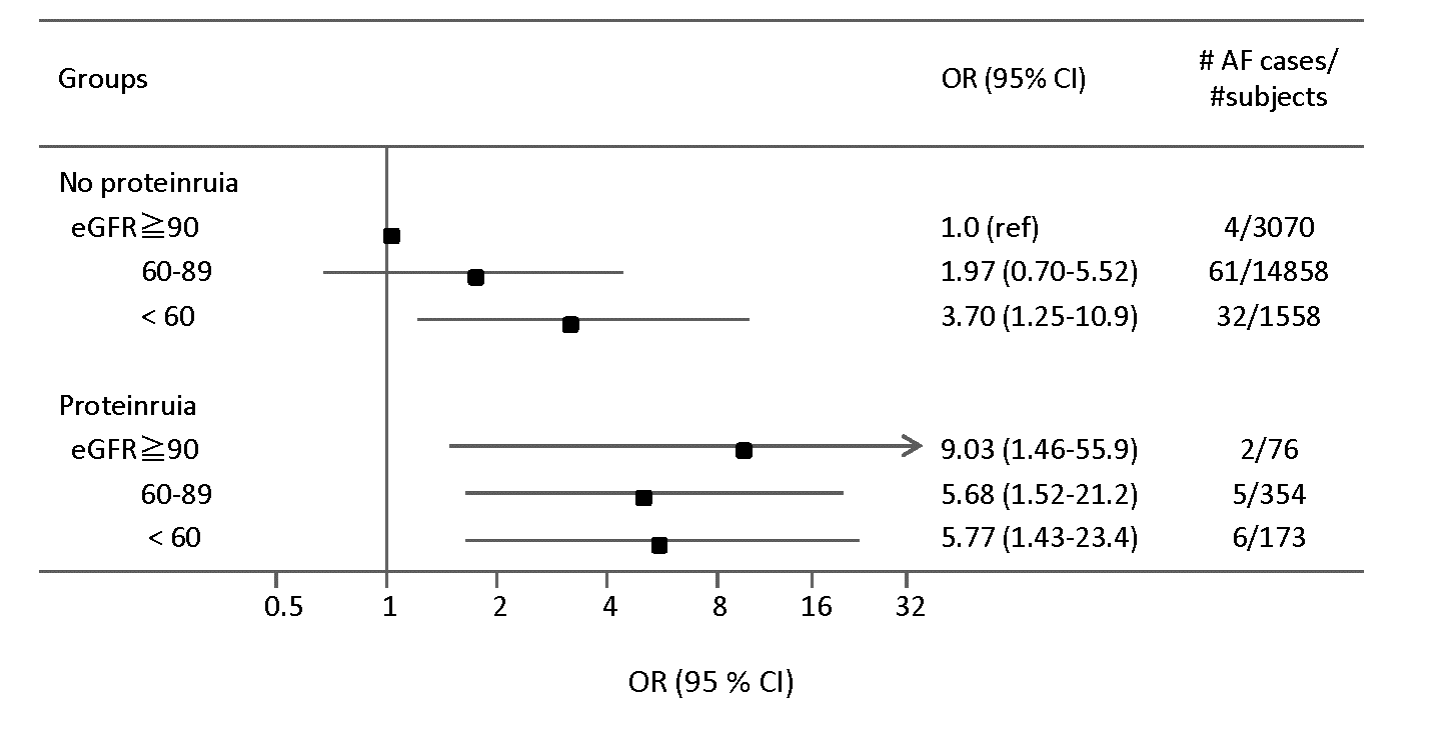
Odds ratios (OR) and 95% confidence intervals (CI) of atrial fibrillation (AF) according to presence of proteinuria and eGFR. Multivariable logistic regression models adjusted for age, gender, hypertension, diabetes mellitus, smoking, and cardiac disease.

 To assess the influence of the presence of proteinuria, we estimated ORs with 6 categories in the models adjusted for covariates as shown in Figure 1. Among the individuals without proteinuria, subjects with < 60 mL▪min^-1^▪1.73m^-2^ were associated with increased OR for AF risk (3.70, [95%CI, 1.25-10.9]). Among the individuals with proteinuria, however, OR for eGFR ≧ 90 mL▪min^-1^▪1.73m^-2^ was higher than those for eGFR 60 to 89 and <60 mL▪min^-1^▪1.73m^-2^, (9.03, [95%CI, 1.46-55.9], 5.68, [95%CI, 1.52-21.2] and 5.77 [95%CI, 1.43, 23.4], respectively). There was no significant interaction between level of eGFR and proteinuria (p=0.531). 

 We also examined the relationship of the reduced eGFR and proteinuria with prevalent AF in men and women separately, and found positive association with AF in both sexes although men were more likely to have AF than women (0.80% vs 0.17%, respectively; p<0.001). Multivariable analysis revealed that an increased OR for AF risk in the subjects with reduced eGFR (<60 mL▪min^-1^▪1.73m^-2^) remains statistically significant after adjusting for age, hypertension, diabetes mellitus, smoking and cardiac disease in men, but not in women (2.23, [95%CI, 1.22-4.07] vs 2.70, [95%CI 0.29-24.65], respectively), probably due to fewer number of AF cases in women (men 99, women 13) in our study population. 

## Discussion

In this cross-sectional, community-based study in which > 20,000 Japanese adults participated, we demonstrated that eGFR <60 mL▪min^-1^▪1.73m^-2^ and proteinuria were separately and strongly associated with an increased prevalent AF independent of established risk factors on multivariable logistic regression analysis. Moreover, we demonstrate that among individuals with proteinuria, the subjects with eGFR≧90 mL▪min^-1^▪1.73m^-2^ are likely to have a similar or even higher risk for AF compared with those with mildly reduced eGFR. These findings are generally consistent with the previous studies demonstrating the impaired kidney function and albuminuria are associated with AF risk, and suggest that treatment and prevention strategies targeting CKD may have potential effect for the prevention of AF in the community. 

### Comparison with the prior studies

To date, the association of CKD with prevalent AF has consistently been reported in three cross-sectional studies [9-11]. For example, in KAMS (Kurashiki City Annual Medical Survey) study [11], enrolling 41,417 subjects living in Kurashiki city, the odd ratio for AF adjusted for variable risk factors was 1.91 (95% confidence interval 1.54 to 2.38, p<0.001) for the low-GFR tertile versus the high tertile. In REGARDS (Reasons for geographic and racial differences in stroke) study [9] enrolling 26,917 participants in United States, the subjects with CKD were more likely to have AF regardless of the severity of CKD. The Heart and Soul study [10] showed that prevalent AF was associated with decreasing GFR in the patients with coronary artery disease (CAD). Although the participants in our cross-sectional study cannot be diagnosed as CKD because we evaluated the kidney function and proteinuria based on one single measurement, our study highlighted the importance of the impaired kidney function and proteinuria as risk factor for AF even in the younger subjects whose prevalence of cardiovascular co-morbidity is significantly less than that in the aforementioned studies (3.2 % in this study). The mean age was 53.2 years in this study and around 70 years in prior three studies. Accordingly, the association between the impaired kidney function and AF may exist among study population with different range of demographic and clinical characteristics of the participants from that evaluated by prior studies. 

### Comparison with the CKD-EPI equation

We categorized eGFR of study population using The MDRD Study equation which has been commonly used to estimate GFR and has been recommended by Kidney Disease: Improving Global Outcomes (KDIGO) clinical practice guidelines for CKD [3]. Recently, Levey et al. developed CKD-EPI (Chronic Kidney Disease Epidemiology Collaboration) equation, which applies different coefficiencies to the identical variables (age, sex, race, and serum creatinine level) used in The MDRD Study equation. Several studies have shown that the CKD-EPI equation was more precise and accurate than The MDRD Study equation, especially at higher GFR [12]. In addition, eGFR computed by the CKD-EPI equation has been proposed to predict risk for adverse outcomes better than The MDRD Study equation in a broad range of populations including Asian population [13]. Accordingly, we separately evaluated the AF risk relationship with eGFR categorized by CKD-EPI equation modified for Japanese [8] and MDRD Study equation. The results between by CKD-EPI equation and by MDRD study equation are almost similar, but the association did not remain significant after adjustment for multiple risk variables and proteinuria. The lower number of subjects with AF among the impaired eGFR (<60 mL▪min^-1^▪1.73m^-2^) subjects in the CKD-EPI equation (24 in the CKD-EPI equation, 38 in The MDRD Study equation) probably makes our analysis underpowered to detect association. 

### Potential mechanisms

Because of the observational nature of our study, the biological relevance of the association between the impaired kidney function and/or proteinuria and prevalent AF remains uncertain. However, taking the low prevalence of obesity, cardiac disease, stroke, and uncontrolled hypertension in our cohort, we do not believe that association between the impaired kidney function and/or proteinuria and AF was due to these substantial confounding factors. Instead, we assume that the impaired kidney function and/or proteinuria *per se* may be a risk factor for AF. This supposition is supported by our multivariable logistic regression analysis indicating that the magnitude of the association between AF and eGFR < 60 mL▪min^-1^▪1.73m^-2^ was attenuated only modestly after adjustment for age, gender and other established risk factors. The same was also true for the association between AF and proteinuria. Therefore these data suggest that traditional risk factors could only partly explain an increased risk for AF in the population with fimpaired kidney function and/or proteinuria in our cohort. 

Question is how the impaired kidney function and/or proteinuria *per se* affects the risk of AF. Several possible mechanisms may account for these associations. First, chronic inflammation may contribute to the increased risk of AF in subject with the impaired kidney function because inflammation has been implicated in both the initiation and maintenance of AF [14]. There have been several population-based studies showing that CRP was independently associated with baseline AF and with the future development of AF [15,16]. In addition, previous study showed that rosuvastatin use was associated with a significant reduction in the risk of incident AF as well as a reduction in hs-CRP levels compared with placebo. These results suggest that inflammatory response elicited in atrial endothelial cells may contribute to AF in the subjects with CKD. Second, because strong evidence indicates that activation of renin-angiotensin-aldosterone system is an important contributor to the genesis of AF, positive association between CKD and AF may be attributed to the unfavorable effects of angiotensin II and aldosterone on atrial structural and electrical remodeling via their pro-fibrotic, pro-inflammatory and pro-oxidative effects [17,18]. Although ARB did not prevent the recurrence of AF in large, randomized prospective study, further studies should be warranted to determine whether early intervention with RAS blockade prevent incident AF in the population at risk for AF. Alternatively, but it is not mutually exclusive, lower eGFR may be a consequence of AF rather than a cause of AF because AF is likely to cause the decline in kidney function owing to a decrease in cardiac output. In fact, Takahashi have reported that kidney function improved after the maintenance of sinus rhythm by successful catheter ablation of AF [19]. 

 With regard to proteinuria, an increased prevalence of AF was apparent in subjects within any categories of eGFR. It is interesting to note that OR in proteinuric subjects with eGFR ≧90 mL▪min^-1^▪1.73m^-2^ appeared to be comparable with or even higher than that in subjects with eGFR 60-89 and <60 mL▪min^-1^▪1.73m^-2^. These findings are also consistent with previous report demonstrating that macroalbuminuria (albumin creatinine ratio ≧ 300 mg/g) was associated with incidence of AF among individuals with eGFR≧90 mL▪min^-1^▪1.73m^-2^, compared with eGFR 60 to 89 mL▪min^-1^▪1.73m-^2^ [20]. There has been a study demonstrating that the burden of AF among individuals with both proteinuria and reduced eGFR may be substantially higher than in those with only 1 of these abnormalities [9], but this study didn’t evaluate the difference between eGFR ≧90 mL▪min^-1^▪1.73m^-2^ and eGFR 60 to 89 mL▪min^-1^▪1.73m^-2^. 

### Limitations

The major limitation of the present study is the cross-sectional observational design. This precludes inferences about a causal relationship between CKD and AF. Prospective studies are required to explain how a declining eGFR or an increasing proteinuria can lead to a progression in AF risk. Second limitation is that prevalent AF was only identified from one single standard 12-lead electrocardiogram, possibly underestimating the true prevalence of paroxysmal AF. Third limitation is the only one measurement of serum creatinine and a urine dipstick protein, which potentially causes measurement bias. Because of this limitation, we cannot refer to subjects with the reduced eGFR and proteinuria as ‘CKD patients’ explicitly defined by KDIGO guidelines [3]. Forth limitation is that we estimated GFR based on levels of serum creatinine, which might be affected by muscle mass or diet independent of GFR. Further studies exploring the comparison between creatinine-based and cystatin C-based eGFR should be performed to determine whether association between eGFR and prevalent AF is similar irrespective of filtration marker used to estimate GFR. Fifth limitation is the use of dipstick to evaluate proteinuria that is not quantitative method. Sixth limitation is the fact that the number of patients with AF was low, probably because of the relatively young population in this study. This might have affected the strength of the observed associations. Lastly, the participants in this study are exclusively Japanese, and it remains to be established whether the results can be generalized to other races and ethnicities. 

## Conclusions

Proteinuria and lower eGFR were separately and independently associated with the prevalence of AF in this large population-based cohort of Japanese adults. Given the growing burden of CKD in general population, additional prospective studies are required to determine the mechanisms responsible for this association, and to help target intervention designed to reduce AF risk in the community. 

## Supporting Information

Table S1 **Odds Ratio (95%CI) for AF According to eGFR levels using the CKD-EPI equation.**
(PPTX)

Table S2 **Odds Ratio (95%CI) for AF According to Presence of Proteinuria after adjusted multivariable and eGFR using the CKD-EPI equation.**
(PPTX)
