# Supplementary material for: Estimated Glomerular Filtration Rate and Proteinuria Are Separately and Independently Associated with the Prevalence of Atrial Fibrillation in General Population
Source: PLoS One. 2013 Nov 6;8(11):e79717. doi: 10.1371/journal.pone.0079717 (PMC3819254; doi:10.1371/journal.pone.0079717)
Supplement: Table S1 — Odds Ratio (95%CI) for AF According to eGFR levels using the CKD-EPI equation. (PPTX) [file pone.0079717.s001.pptx]

## Slide 1
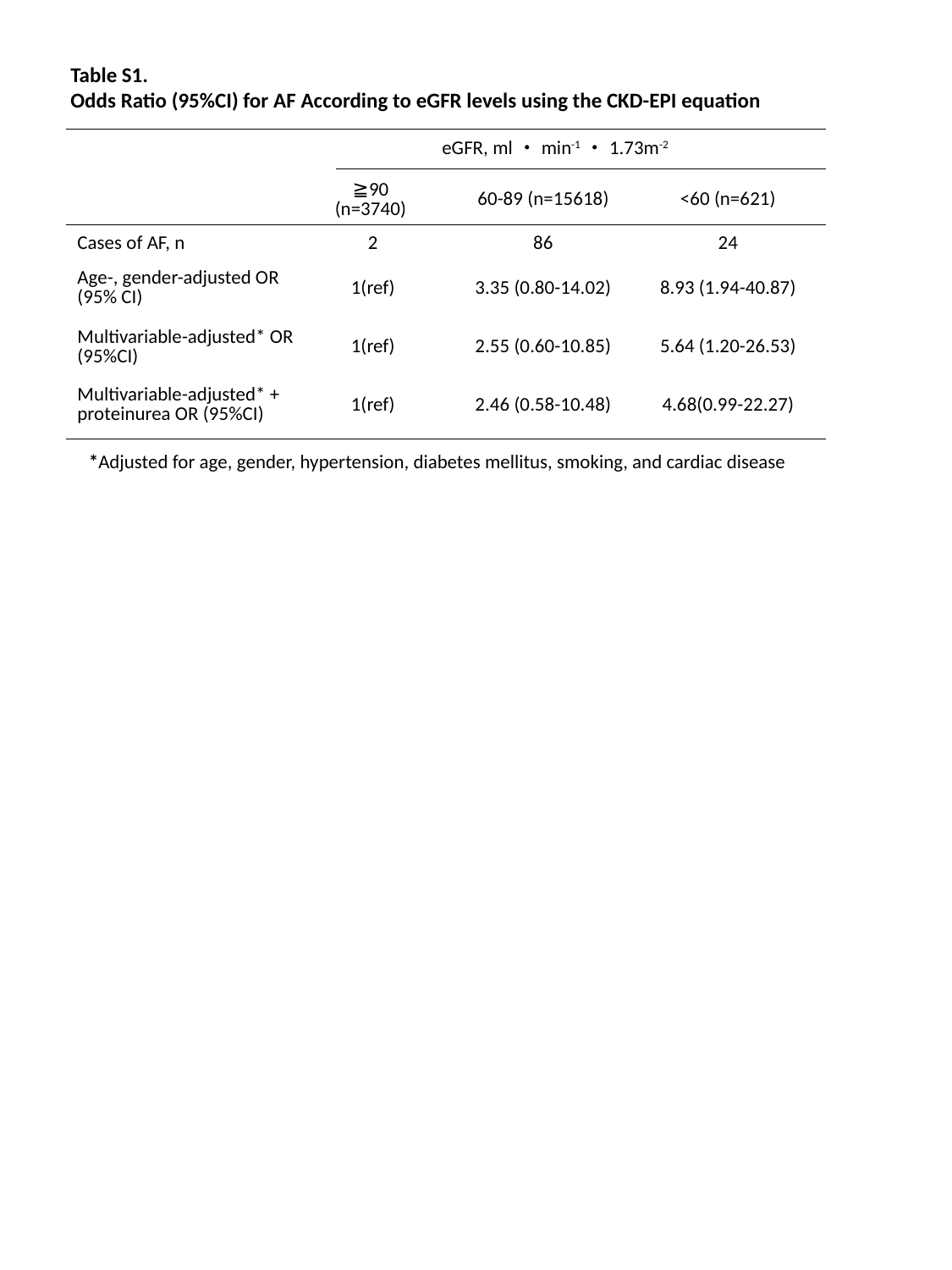

Table S1.
Odds Ratio (95%CI) for AF According to eGFR levels using the CKD-EPI equation
eGFR, ml・min-1・1.73m-2
| | ≧90 (n=3740) | 60-89 (n=15618) | <60 (n=621) |
| --- | --- | --- | --- |
| Cases of AF, n | 2 | 86 | 24 |
| Age-, gender-adjusted OR (95% CI) | 1(ref) | 3.35 (0.80-14.02) | 8.93 (1.94-40.87) |
| Multivariable-adjusted\* OR (95%CI) | 1(ref) | 2.55 (0.60-10.85) | 5.64 (1.20-26.53) |
| Multivariable-adjusted\* + proteinurea OR (95%CI) | 1(ref) | 2.46 (0.58-10.48) | 4.68(0.99-22.27) |
*Adjusted for age, gender, hypertension, diabetes mellitus, smoking, and cardiac disease
