# Supplementary material for: Estimated Glomerular Filtration Rate and Proteinuria Are Separately and Independently Associated with the Prevalence of Atrial Fibrillation in General Population
Source: PLoS One. 2013 Nov 6;8(11):e79717. doi: 10.1371/journal.pone.0079717 (PMC3819254; doi:10.1371/journal.pone.0079717)
Supplement: Table S2 — Odds Ratio (95%CI) for AF According to Presence of Proteinuria after adjusted multivariable and eGFR using the CKD-EPI equation. (PPTX) [file pone.0079717.s002.pptx]

## Slide 1
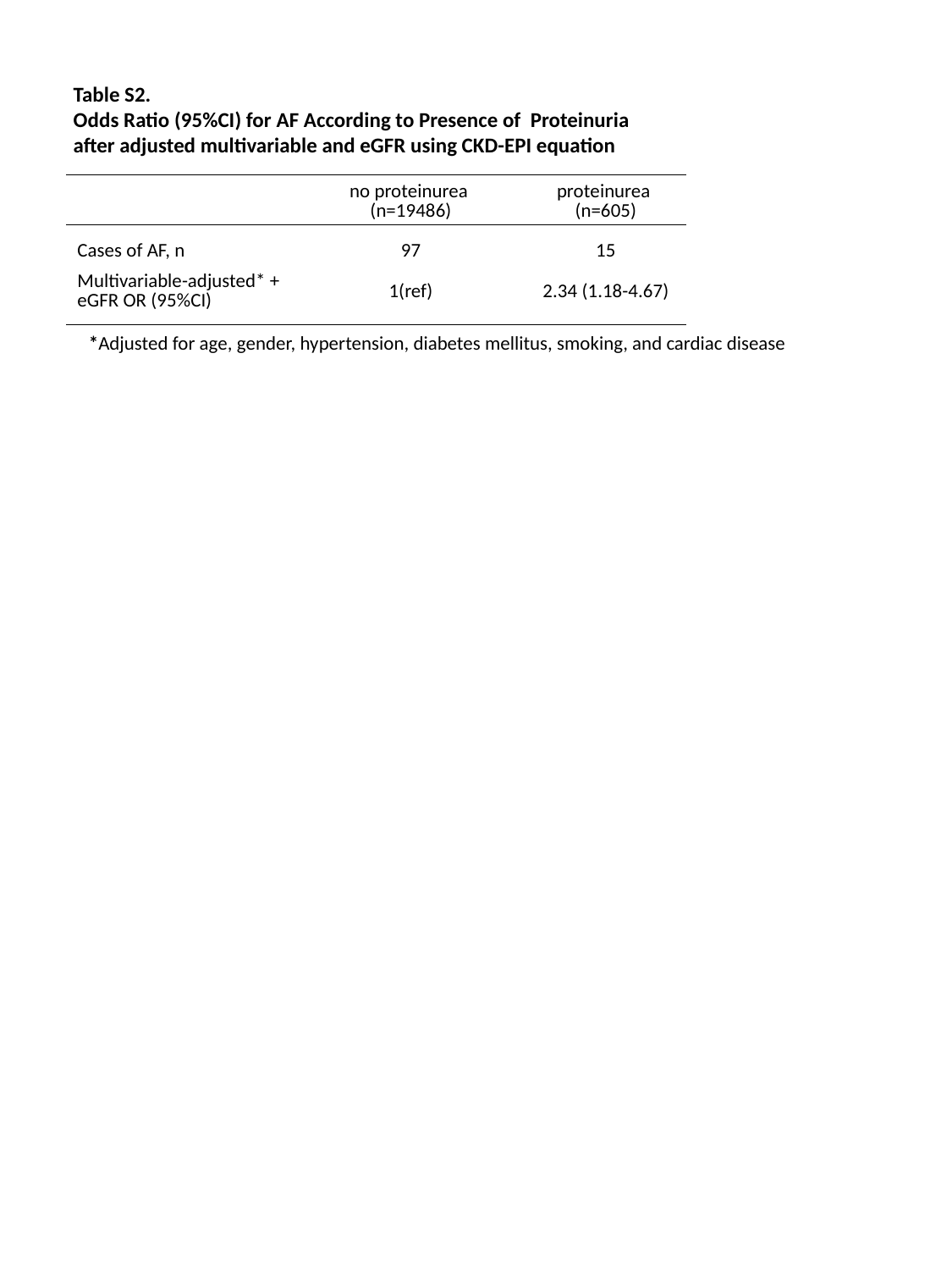

Table S2.
Odds Ratio (95%CI) for AF According to Presence of Proteinuria
after adjusted multivariable and eGFR using CKD-EPI equation
| | no proteinurea (n=19486) | proteinurea (n=605) |
| --- | --- | --- |
| Cases of AF, n | 97 | 15 |
| Multivariable-adjusted\* + eGFR OR (95%CI) | 1(ref) | 2.34 (1.18-4.67) |
*Adjusted for age, gender, hypertension, diabetes mellitus, smoking, and cardiac disease
